# Latent class profile model with time-dependent covariates: a study on symptom patterning of patients for head and neck cancer

**DOI:** 10.1080/02664763.2024.2435997

**Published:** 2024-12-16

**Authors:** Jung Wun Lee, Hayley Dunnack Yackel

**Affiliations:** a Department of Biostatistics, Harvard T.H. Chan School of Public Health, Boston, MA, USA; b Hartford HealthCare Cancer Institute, Hartford, CT, USA

**Keywords:** EM algorithm, longitudinal data, categorical data, head and neck cancer, chemoradiation treatment

## Abstract

The latent class profile model (LCPM) is a widely used technique for identifying distinct subgroups within a sample based on observations' longitudinal responses to categorical items. This paper proposes an expanded version of LCPM by embedding time-specific structures. Such development allows analysts to investigate associations between latent class memberships and time-dependent predictors at specific time points. We suggest a simultaneous estimation of latent class measurement parameters via the expectation-maximization (EM) algorithm, which yields valid point and interval estimators of associations between latent class memberships and covariates. We illustrate the validity of our estimation strategy via numerical studies. In addition, we demonstrate the novelty of the proposed model by analyzing the head and neck cancer data set.

## 1. Introduction

Head and neck cancer (HNC) is a challenging disease that may entail multiple combined and complex treatments. Due to its tumor location, patients with HNC may suffer from adverse effects, such as mucositis, lack of appetite, xerostomia, altered taste and flavor sensations, fatigue, and pain [26,27]. In this sense, it is important to examine the trends of symptom expression to improve symptom management in this population [29,30]. Recently, Yackel *et al*. [30] performed a retrospective, longitudinal study by collecting records of patients who underwent concurrent chemotherapy (CT) and radiation therapy (RT) throughout six-week treatment periods (hereafter, we denote the collected records as HNC data set).

Yackel *et al*. [30] employed the latent transit model (LTM) to identify four latent classes of patients based on unpleasant symptom patterns and transitional matrices that describe how patients' latent class memberships change over treatment periods. LTM is one type of Hidden-Markov model (HMM) specialized for multivariate categorical longitudinal outcomes [9,17]. Based on the first-order Markov chain assumption, the LTM identifies two components of latent structures: the distribution of latent classes at the baseline and the transition matrices that describe transitions of latent class memberships between adjacent time points. For each time point, subject-level covariates can be embedded in the model and subject-level conditional probabilities of latent class memberships can be calculated.

When the sample size is small while the number of periods *T* is large, the estimation of transition probabilities can be unstable. This is one of the main issues in Yackel *et al*. [30], where the sample size is *N* = 275, and the six outcome variables were repeatedly measured across six periods. The four-class LTM defined on six-time periods required 104 parameters to be estimated ( 6×4=24 for four-class measurement and 5×4×4=80 for five transition matrices). The estimation algorithm did not converge, and the fitted model was not locally identifiable, so the authors simplified the analysis model by reducing the number of periods to 3 (that is, by discarding the data from the second, fourth, and fifth weeks).

Another limitation of using LTM is that it only discovers time-specific transition patterns of latent classes and does not identify trajectory patterns of subjects accounting for the entire time sequence. Namely, estimated quantities from LTM are limited to latent classes at baseline and their transitions between two adjacent time periods, and does not include ‘trajectories’ over entire time periods. Such aspect is not a particular disadvantage of LTM, but rather a fundamental limitations of all HMM based frameworks, including Altman [1], Bartolucci *et al*. [5,6]. Consequently, when a research interest lies on examining the trends of symptom expression over entire study periods, a HMM-based methodology is not appropriate.

This paper aims to overcome the limitations of Yackel *et al*. [30] by suggesting an enhanced categorical latent variable model for longitudinal data. Our proposed model employs the latent class profile model (LCPM) [7,19,20] to avoid the first-order Markov chain assumption. Instead, LCPM uses a secondary categorical latent variable whose category represents a sequential pattern of latent classes over time. Such a two-level structure allows LCPM to identify latent longitudinal patterns of latent class using fewer assumptions and a smaller number of parameters.

A statistical novelty of this paper is the proposal of including time-varying predictors in the conventional latent class profile model by parameterizing secondary measurement parameters as functions of individual-level time-dependent predictors. The proposed LCPM provides subject-specific probabilities of latent class memberships for a specific time, which may improve the classification accuracy of the LCPM. We suggest a one-step simultaneous estimation strategy that estimates the measurement parameters (i.e. parameters related to class and profile identification) and structural parameters (i.e. parameters that describe the associations between time-dependent covariates on the latent class memberships at a specific time and associations between time-independent covariates and the latent profile memberships) simultaneously.

The structure of this paper is as follows. Section 2 reviews the conventional LTM and LCPM suggested by Chung *et al*. [7] and proposes the expanded LCPM by embedding subject-specific time-dependent covariates. Section 3 introduces an estimation strategy based on the EM algorithm and demonstrates its performance via simulation studies. Section 5 presents the application of the proposed LCPM to the HNC data set. Section 6 discusses the conclusions and future research goals.

## 2. Methods

### 2.1. Latent transition model (LTM)

The latent transition model postulates a sequence of categorical latent variables that provides a set of partitions (i.e. latent classes) of the target population at the baseline and describes the transition of class memberships over time [9]. Let $Y_{\mathrm{it}}=[Y_{i1t},\ldots ,Y_{\mathrm{iMt}}]$ be a vector of *M* categorical measurement variables from *i*th subject at time *t*, where t=1,…,T is a discrete time sequence. Each $Y_{\mathrm{imt}}$ has one of $1,\ldots ,r_{m}$ possible outcomes for all *t*, so the number of possible response patterns of each subject is $\prod_{m=1}^{M}r_{m}^{T}$. For example, if four binary indicators consist of an outcome vector across three time periods, then $(2^{4})=16$ patterns exist in the baseline and $(2^{4}{)}^{3}=4,096$ possible patterns across three periods. Such a large number of patterns are summarized into *K* numbers of patterns by introducing a discrete latent variable $C_{1}$ with *K* categories and *K* by *K* transition matrices between adjacent time periods. In such a way, subjects with the same latent class memberships are homogeneous in their response patterns and heterogeneous if they belong to different latent classes. In addition, we assume that the outcome variables are conditionally independent given latent class membership (local independence) [3,12]. Under such assumptions, we can construct the joint distribution of $Y_{i}=[Y_{i1},\ldots ,Y_{\mathrm{iT}}]$ as

(1) $$\begin{array}{rl} P(Y_{i}|x_{i},z_{i}) & =\sum_{c_{1}=1}^{K}\cdots \sum_{c_{T}=1}^{K}P(Y_{i1},\ldots ,Y_{\mathrm{iT}},C_{1},\ldots ,C_{T}|x_{i},z_{i})\\ & =\sum_{c_{1}=1}^{K}\cdots \sum_{c_{T}=1}^{K}P(Y_{i1},\ldots ,Y_{\mathrm{iT}}|C_{1},\ldots ,C_{T})P(C_{1},\ldots ,C_{T},x_{i},z_{i})\\ & =\sum_{c_{1}=1}^{K}\cdots \sum_{c_{T}=1}^{K}P(C_{1}|x_{i},z_{i1})\prod_{t=2}^{T}P(C_{t}|C_{t-1},z_{\mathrm{it}})\prod_{t=1}^{T}P(Y_{\mathrm{it}}|C_{t}=c). \end{array}$$

In Equation (1), $x_{i}=[x_{i1},\ldots ,x_{\mathrm{iP}}{]}^{\prime }$ denotes a vector of subject-level baseline predictor, and $z_{\mathrm{it}}=[z_{i1t},\ldots ,z_{\mathrm{iQt}}{]}^{\prime }$ is a vector of time-dependent predictors. Also, the time-invariant assumption has been imposed such that $P(Y_{\mathrm{it}}|C_{t}=c_{t})$ is equal across all t=1,…,T [9]. This assumption can be tested via the likelihood ratio test, as suggested in [9,19]. $P(C_{t}|C_{t-1},z_{\mathrm{it}})$ denotes transition probabilities of *K* latent classes between time periods *t*−1 and *t*, which requires K(K+1)/2 parameters if unstructured. Note that the number of latent class *K* is unknown and should be determined by an analyst. For more details on determining *K*, see Jeon *et al*. [16], Nylund *et al*. [21].

### 2.2. Latent class profile model (LCPM)

The LCPM is another longitudinal model for multivariate categorical outcomes that tracks sequential patterns of a categorical latent variable over time. Instead of tracking transitions of baseline latent classes over time, LCPM is devised to find the latent trajectories of subjects based on their response patterns using two layers of categorical latent variables. [7,19,20]. In the first layer, a vector of categorical latent variables $C=[C_{1},\ldots ,C_{T}]$ is estimated based on $Y_{i}$, where each $Y_{t}$ is related to $C_{t}$ only. Next, the joint distribution of C is decomposed into the product of the marginal density of $C_{t}$ conditioning on *U*. Consequently, the joint distribution of $Y_{i}$ is written as

(2) $$\begin{array}{rl} P(Y_{i}|x_{i}) & =\sum_{u=1}^{S}\sum_{c_{1}=1}^{K}\cdots \sum_{c_{T}=1}^{K}P(Y_{i},C=c,U=u|x_{i})\\ & =\sum_{u=1}^{S}\sum_{c_{1}=1}^{K}\cdots \sum_{c_{T}=1}^{K}P(U=u|x_{i})P(C=c|U=u)P(Y_{i}=y_{i}|C=c)\\ & =\sum_{u=1}^{S}\sum_{c_{1}=1}^{K}\cdots \sum_{c_{T}=1}^{K}P(U=u|x_{i})\prod_{t=1}^{T}\left[P(C_{t}=c_{t}|u)\prod_{m=1}^{M}P(Y_{\mathrm{imt}}|c_{t})\right]. \end{array}$$

In the first layer, subject-level response patterns at time *t* are summarized into *K* categorical latent variable $C_{t}$ (i.e. latent class variable), so subjects with the same class membership are homogeneous in their response patterns. In the second layer, the joint distribution of $\left[C_{1},\ldots ,C_{T}\right]$ is summarized into *S* categorical latent variable *U* (i.e. latent profile variable) so that subjects with the same profile membership share the same class membership patterns over time [7,19,20].

The construction of the LCPM requires the following assumptions: (1) the latent profile membership is related to the manifest items only through the class membership of each latent variable at each time wave. (2) the response variables at time *t* (i.e. $Y_{t}=[Y_{1t},\ldots ,Y_{\mathrm{Mt}}]$) become mutually independent when the latent class membership $c_{t}$ is known, (3) latent class variable $C=[C_{1},\ldots ,C_{T}]$ become mutually independent when the latent class profile membership *U* = *u* is known, and (4) primary measurement parameters probabilities $P(Y_{\mathrm{mt}}=k|c_{t})$ are invariant to the individual covariates $x_{i}$. Assumptions (2) and (3) indicate local independence, simplifying the likelihood function of complex latent structures. Assumption (4) is the conditional independence to preserve the consistent interpretation of latent classes [2]. Finally, Chung *et al*. [7] suggests three parameter types to specify the likelihood function of LCPM in Equation (2).

1.
$\rho_{\mathrm{mk}|c_{t}}=P(Y_{\mathrm{mt}}=k|c_{t})$ is a primary measurement parameter, which denotes the probability of a response variable $y_{\mathrm{mt}}$ has value *k* given that the latent class membership at time *t* is $c_{t}$.
2.
$\eta_{c_{t}|u}^{\left(t\right)}=P(C_{t}=c_{t}|U=u)$ is a secondary measurement parameter, which denotes the conditional probability of belonging to class $c_{t}$ at time *t* given that a latent profile membership is *U* = *u*.
3.
$\gamma_{u}=P(U=u)$ denotes the probability that a subject individual belongs to the latent profile *u*.

The primary measurement parameter ρ describes latent class structures at a specific time. The secondary measurement parameter η describes distributions of latent classes at a specific time and characterizes latent profiles. Finally, the prevalence parameter γ represents the proportion of latent profiles. These parameters only reflect the contribution of outcome variables on the likelihood function and do not consider covariate effects. Chung *et al*. [7] replaces the prevalence parameter γ with a logistic function of individual-level covariates as

(3) $$\gamma_{u}(x_{i})=P(U=u|x_{i})=\frac{\exp(x_{i}^{\prime }{\text{β}}_{u})}{\sum_{s=1}^{S}\exp(x_{i}^{\prime }{\text{β}}_{s})},\;{\text{β}}_{u}\in R^{P},\;{\text{β}}_{1}=0,\;u=1\ldots ,S.$$

In Equation (3), ${\text{β}}_{u}$ represents associations between demographic factors $x_{i}^{\prime }$ and *u*th latent profile using multinomial logistic regression. In the next section, we expand the LCPM by replacing the secondary measurement parameter $\eta_{c_{t}|u}^{\left(t\right)}$ with the function of time-dependent covariate $z_{\mathrm{it}}$. For more details on conventional LCPM, see Chung *et al*. [7], Lee and Chung [19], Lee *et al*. [20].

### 2.3. Latent class profile model with time-dependent covariate

**Figure 1.**
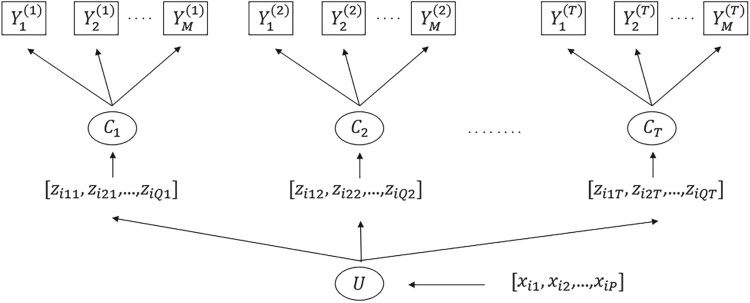
A diagram of multivariate latent class profile model with time-dependent (**Z**) and independent (**X**) covariates.

A novelty of this paper is to expand the LCPM in Equation (2) by considering the effect of time-dependent covariates on the distribution of latent classes. Let $z_{\mathrm{it}}=[z_{i1t},\ldots ,z_{\mathrm{iQt}}{]}^{\prime }$ be a vector of time-dependent covariates of a subject *i* at time *t*, which may influence the probability of belonging to the latent class membership $C_{t}$ at time *t*. Figure 1 illustrates the diagram of the proposed LCPM.

Let $\alpha_{c_{t}|u}^{\left(t\right)}=[\alpha_{1c_{t}|u}^{\left(t\right)},\ldots ,\alpha_{Qc_{t}|u}^{\left(t\right)}{]}^{\prime }$ be the regression coefficients that specify association between latent class membership $c_{t}$ given latent profile membership *u* at time *t*. Since each latent variable has *K* categories, we use the multinomial logit link as

(4) $$\eta_{c_{t}|u}^{\left(t\right)}(z_{\mathrm{it}})=\frac{\exp(z_{\mathrm{it}}^{\prime }\alpha_{c_{t}|u}^{\left(t\right)})}{\sum_{k=1}^{K}\exp(z_{\mathrm{it}}^{\prime }\alpha_{k|u}^{\left(t\right)})},\;\alpha_{c_{t}|u}^{\left(t\right)}\in R^{Q},\;\alpha_{1|u}^{\left(t\right)}=0,\;c_{t}=1\ldots ,K,\;u=1,\ldots ,S.$$

Consequently, the joint distribution of $Y_{i}$ given $\left[x_{i},z_{i}\right]$ can be written as

(5) $$\begin{array}{rl} P(Y_{i}|x_{i},z_{i}) & =\sum_{u=1}^{S}\sum_{c_{1}=1}^{K}\cdots \sum_{c_{T}=1}^{K}P(Y_{i},C=c,U=u|x_{i},z_{i})\\ & =\sum_{u=1}^{S}\sum_{c_{1}=1}^{K}\cdots \sum_{c_{T}=1}^{K}P(U=u|x_{i})P(C=c|U=u,z_{\mathrm{it}})P(Y_{i}|C=c)\\ & =\sum_{u=1}^{S}\sum_{c_{1}=1}^{K}\cdots \sum_{c_{T}=1}^{K}P(U=u|x_{i})\prod_{t=1}^{T}\left[P(C_{t}=c_{t}|u,z_{\mathrm{it}})\prod_{m=1}^{M}P(Y_{\mathrm{imt}}|c_{t})\right]. \end{array}$$

Using the complete data likelihood $L^{*}(u,c|y_{i},x_{i},z_{i})$ and the observed data likelihood $L(y_{i},x_{i},z_{i})$ can be written as

(6) $$\begin{array}{rl} L^{*}(u,c|y_{i},x_{i},z_{i}) & =\gamma_{u}(x_{i})\prod_{t=1}^{T}\left[\eta_{c_{t}|u}^{\left(t\right)}(z_{\mathrm{it}})\prod_{m=1}^{M}\prod_{k=1}^{r_{m}}\rho_{\mathrm{mk}|c_{t}}^{I(y_{\mathrm{mt}}=k)}\right],\\ L(y_{i},x_{i},z_{i}) & =\sum_{u=1}^{S}\sum_{c_{1}=1}^{K}\cdots \sum_{c_{T}=1}^{K}\gamma_{u}(x_{i})\prod_{t=1}^{T}\left[\eta_{c_{t}|u}^{\left(t\right)}(z_{\mathrm{it}})\prod_{m=1}^{M}\prod_{k=1}^{r_{m}}\rho_{\mathrm{mk}|c_{t}}^{I(y_{\mathrm{mt}}=k)}\right]. \end{array}$$

where a time-specific subscript *t* is aggregated so that $y_{i}=[y_{i1},\ldots ,y_{\mathrm{iT}}]$ and $z_{i}=[z_{i1},\ldots ,z_{\mathrm{iT}}]$. Also, I(A) denotes the indicator function, which takes the value 1 if an event *A* is true and 0 otherwise.

As shown in Equation (4), the immediate advance from the standard LCPM to the proposed LCPM is the consideration of time-dependent predictors on the distributions of latent classes. In the proposed LCPM, the secondary measurement parameters η are expanded as functions of time-dependent predictors z with structural parameters α. Consequently, the parameters of the proposed LCPM consist of three types: outcome measurement parameters ρ, structural parameters β for time-independent predictors, and structural parameters α for time-independent predictors.

When fitting the LCPM, it is practical to assume that the primary measurement parameters are equal across time so that $P(Y_{\mathrm{mkt}}=k|c_{t})$ is invariant to *t*. Such constraint implies $\rho_{\mathrm{mk}|c_{t}}=\rho_{\mathrm{mk}|c}$ for all subscripts and t=1,…,T, which reduces the complexity of the model and yields the consistent interpretations on latent class memberships over time. By doing so, the LCPM can keep track of sequential patterns of latent classes at the initial time point. Such assumption can be tested via the likelihood ratio test [19] or the Pearson-residual statistics [20]. Lastly, the number of latent classes *K* and the profiles *S* should be determined by an analyst. We discuss the model selection procedure in Section 3.3.

## 3. Estimation and inference

### 3.1. Recursive expectation-maximization algorithm

The parameter estimation in the LCPM is one type of missing data problem because the latent class memberships over time and the latent profile memberships cannot be determined from the observed data. In this sense, the EM algorithm can be one strategy to obtain the maximum likelihood (ML) estimates of model parameters. The conventional EM algorithm implements the Expectation step (E-step) and Maximization step (M-step) for each iteration and repeats these steps until the solutions satisfy the convergence threshold. Specifically, We calculate the expectation of the conditional distributions of latent variables given the response variables in E-step, and we update the parameter estimates using the estimators that maximize the expectation. The rest of the section provides the details of the EM algorithm implementation tailored for LCPM with time-dependent covariates.

*E-step*. We calculate the full conditional probability of latent variables given the *i*th observed responses and covariates as

(7) $$\begin{array}{rl} \theta_{i(u,c)} & =P(U=u,C_{1}=c_{1},\ldots ,C_{T}=c_{T}|y_{i},x_{i},z_{i})\\ & =\frac{\gamma_{u}(x_{i})\prod_{t=1}^{T}\left[\eta_{c_{t}|u}^{\left(t\right)}(z_{\mathrm{it}})\prod_{m=1}^{M}\prod_{k=1}^{r_{m}}\rho_{\mathrm{mk}|c_{t}}^{I(y_{\mathrm{mt}}=k)}\right]}{\sum_{u=1}^{S}\sum_{c_{1}=1}^{K}\cdots \sum_{c_{T}=1}^{K}\gamma_{u}(x_{i})\prod_{t=1}^{T}\left[\eta_{c_{t}|u}^{\left(t\right)}(z_{\mathrm{it}})\prod_{m=1}^{M}\prod_{k=1}^{r_{m}}\rho_{\mathrm{mk}|c_{t}}^{I(y_{\mathrm{mt}}=k)}\right]}, \end{array}$$

where subscript ranges are i=1,…,N, u=1,…,S, $c_{t}=1,\ldots ,K$, and t=1,…,T. Since all latent variables $c=[c_{1},\ldots ,c_{T}]$ are categorical, the conditional distribution of each latent variable given [y,z,x] follows a multinomial distribution. Consequently, the log-complete data likelihood and its expected value can be written as

(8) $$\begin{array}{rl} l^{*}(u,c|y_{i},x_{i},z_{i}) & =\sum_{i=1}^{N}\sum_{t=1}^{T}\sum_{m=1}^{M}\sum_{k=1}^{r_{m}}I(C_{t}=c_{t})I(y_{\mathrm{imt}}=k)\log\rho_{\mathrm{mk}|c_{t}}\\ & \;+\sum_{i=1}^{N}\sum_{t=1}^{T}I(U_{i}=u,C_{t}=c_{t})\log\eta_{c_{t}|u}^{\left(t\right)}(z_{\mathrm{it}})\\ & \;+\sum_{i=1}^{N}I(U_{i}=u)\log\gamma_{u}(x_{i}),\\ E\left(l^{*}(u,c|y_{i},x_{i},z_{i})\right) & =\sum_{i=1}^{N}\sum_{t=1}^{T}\sum_{m=1}^{M}\sum_{k=1}^{r_{m}}\theta_{i(c_{t})}I(y_{\mathrm{imt}}=k)\log\rho_{\mathrm{mk}|c_{t}}\\ & \;+\sum_{i=1}^{N}\sum_{t=1}^{T}\theta_{i(u,c_{t})}\log\eta_{c_{t}|u}^{\left(t\right)}(z_{\mathrm{it}})+\sum_{i=1}^{N}\theta_{i(u)}\log\gamma_{u}(x_{i}), \end{array}$$

where the marginal conditional probabilities in Equation (8) can be calculated as

(9) $$\begin{array}{rl} \theta_{i(u,c_{t})} & =\sum_{c_{1}=1}^{K}\cdots \sum_{c_{t-1}=1}^{K}\sum_{c_{t+1}=1}^{K}\cdots \sum_{c_{T}=1}^{K}\theta_{i(u,c)},\\ \theta_{i(u)} & =\sum_{c_{t}=1}^{K}\theta_{i(u,c_{t})},\;\theta_{i(c_{t})}=\sum_{u=1}^{S}\theta_{i(u,c_{t})}. \end{array}$$

As shown in Equations (8) and (9), the full conditional probability $\theta_{i(u,c)}$ plays a key role in E-step. Unfortunately, the computational cost of Equation (7) is not negligible (especially when the number of time stage *T* is large) because its dimension is $K^{T}\times S$ increases exponentially for *T*. Consequently, we use the recursive formula and avoid such heavy computational costs [4,20].

The key idea of the forward-backward algorithm is to decompose the conditional probability $P(Y,C_{t}=c_{t}|U=u,x,z_{t})$ into a forward probability $P(Y_{1},\ldots ,Y_{t},C_{t}=c_{t}|u,x,z)$ and a backward probability $P(Y_{t+1},\ldots ,Y_{T},U=u|x,z_{t})$. Let $\psi_{\mathrm{it}}(u,c_{t},z_{t})$ and $\lambda_{\mathrm{it}}(u,z_{t})$ represent the forward and backward probabilities of *i*th observation, respectively. Explicit forms of $\psi_{\mathrm{it}}(u,c_{t})$ and $\lambda_{\mathrm{it}}(u)$ under LCPM are written as

(10) $$\begin{array}{rl} \psi_{\mathrm{it}}(u,c_{t},z_{\mathrm{it}}) & =P(Y_{1}=y_{1},\ldots ,Y_{t}=y_{t},C_{t}=c_{t}|u,z_{i1},\ldots ,z_{\mathrm{it}})\\ & =\left\{ \begin{array}{ll} \eta_{c_{t}|u}^{\left(t\right)}(z_{\mathrm{it}})\prod_{m=1}^{M}\prod_{k=1}^{r_{m}}\rho_{\mathrm{mk}|c_{t}}^{I(y_{\mathrm{imt}}=k)}\sum_{c_{t-1}=1}^{K}\psi_{\mathrm{it}}(u,c_{t-1},z_{\mathrm{it}-1}), & \mathrm{if}\;t\geq 2,\\ \eta_{c_{1}|u}^{\left(1\right)}(z_{i1})\prod_{m=1}^{M}\prod_{k=1}^{r_{m}}\rho_{\mathrm{mk}|c_{1}}^{I(y_{\mathrm{im}1}=k)}, & \mathrm{if}\;t=1, \end{array}\right.\\ \lambda_{\mathrm{it}}(u,z_{\mathrm{it}}) & =P(Y_{t+1}=y_{t+1},\ldots ,Y_{T}=y_{T}|u,z_{i(t+1)},\ldots ,z_{\mathrm{iT}})\\ & =\left\{ \begin{array}{ll} \;\sum_{c_{t+1}=1}^{K}\eta_{c_{t+1}|u}^{\left(t+1\right)}(z_{i(t+1)})\;\prod_{m=1}^{M}\prod_{k=1}^{r_{m}}\rho_{\mathrm{mk}|c_{\left(t+1\right)}}^{I(y_{\mathrm{im}(t+1)}=k)}\lambda_{i(t+1)}(u,z_{i(t+1)}). & \\ 1, & \mathrm{if}\;t=T. \end{array}\right. \end{array}$$

The forward and backward probabilities allow us to obtain the posterior probabilities of latent class membership $c_{t}$ at a single time point *t* without considering the combination of whole latent variable sequences. Consequently, the conditional probability of latent class memberships at stage *t* can be written as

(11) $$\theta_{i(u,c_{t})}=\frac{\gamma_{u}(x_{i})\psi_{\mathrm{it}}(u,c_{t},z_{\mathrm{it}})\lambda_{\mathrm{it}}(u,,z_{\mathrm{it}})}{\sum_{u=1}^{S}\sum_{c_{1}=1}^{K}\cdots \sum_{c_{T}=1}^{K}\gamma_{u}(x_{i})\psi_{\mathrm{iT}}(u,c_{T},z_{\mathrm{iT}})}.$$

The dimension of the posterior probability in Equation (11) is K×S, which is noticeably reduced from $K^{T}\times S$. Through simulation studies, Lee *et al*. [20] have shown that the recursive algorithm significantly reduces the computational time.

*M-step*. The M-step maximizes the expected complete-data likelihood with respect to the model parameters. Since the sum of parameters that are used in measuring each latent variable is constrained to be 1 (for instance, $\sum_{u=1}^{S}\gamma_{u}=1$), we use the Lagrange multiplier to maximize the expectation under such constraints. The resulting parameter estimators for the LCPM without covariates are as

(12) $${\hat{\gamma }}_{u}=\frac{1}{N}\sum_{i=1}^{N}\theta_{i(u)},\;{\hat{\eta }}_{c_{t}|u}^{\left(t\right)}=\frac{\sum_{i=1}^{N}\theta_{i(u,c_{t})}}{\sum_{i=1}^{N}\theta_{i(u)}},\;{\hat{\rho }}_{\mathrm{mk}|c_{t}}=\frac{\sum_{i=1}^{N}\theta_{i(c_{t})}I(Y_{\mathrm{imt}}=k)}{\sum_{i=1}^{N}\theta_{i(c_{t})}}.$$

Note that Equation (12) applies only when an analyst does not consider individual-level covariates in the LCPM. We propose a tailored EM algorithm by embedding two Newton-Raphson algorithms in the M-step to simultaneously update structural parameters for time-dependent and independent predictors. While standard and proposed EM algorithms share a common E-step, their M-steps are distinguished in the form of maximizer. Specifically, the maximizer of α the proposed EM algorithm does not have a closed form, while the secondary measurement parameter η in the standard EM algorithm has it. Consequently, in the proposed EM algorithm, we run the Newton-Raphson algorithm on [β,α] while conditioning on other parameter estimates obtained from the previous iteration [3,24].

Let $S_{\text{β}}$ and $S_{\alpha }$ be the first derivatives and let $H_{\text{β}}$ and $H_{\alpha }$ be the second derivatives of the log-observed data likelihood in Equation (8) with respect to β and α, respectively. We can update the regression coefficients via the Newton-Raphson algorithm as follows:

(13) $${\text{β}}^{\left(l+1\right)}={\text{β}}^{\left(l\right)}-H_{{\text{β}}^{\left(l\right)}}^{-1}S_{{\text{β}}^{\left(l\right)}},\;\alpha^{\left(l+1\right)}=\alpha^{\left(l\right)}-H_{\alpha^{\left(l\right)}}^{-1}S_{\alpha^{\left(l\right)}},\;l=0,1,2,\ldots ,$$

and details of the second derivatives of the log-observed data likelihood with respect to β and α are shown in the Appendix. In such a way, the EM algorithm for LCPM with time-dependent/independent predictor(s) can be implemented as follows:

1. Let $\Theta^{\left(l\right)}=[\rho^{\left(l\right)},\alpha^{\left(l\right)},{\text{β}}^{\left(l\right)}]$ be the parameter values at *l*th iteration.
2. Implement E-step in Equation (11) at current values $\Theta^{\left(l\right)}$.
3. Implement M-step in Equation (11) and update $\rho^{\left(l+1\right)}$.
4. Implement Newton-Raphson in Equation (13) and update $\left[{\text{β}}^{\left(l+1\right)},\alpha^{\left(l+1\right)}\right]$.
5. Repeat steps 2.∼4. until the algorithm converges.

It is well known that a solution of the EM algorithm may fall into local maxima of the likelihood function [11,28]. To avoid this, an analyst must try many initial values and choose the one with the highest likelihood as the final solution. Properties of the EM estimates will be further discussed in Section 3.2.

If some individuals contain nonresponses, the maximizer for the primary measurement parameter in Equation (12) should be modified. Let $O_{\mathrm{mt}}$ be indices of individuals who responded to $Y_{\mathrm{mt}}$ and $O_{\mathrm{mt}}^{c}$ be indices of individuals who did not. The posterior probability $\theta_{i(c_{t})}^{\mathrm{obs}}$ is calculated based on available cases only. Next, the additional term is included in primary measurement parameter estimators to incorporate missing information from individuals who are excluded in calculating $\theta_{i(c_{t})}^{\mathrm{obs}}$ due to nonresponses to $Y_{\mathrm{imt}}$. Under the missing at random (MAR) assumption, the contribution of individuals in $O_{\mathrm{mt}}^{c}$ can be specified via ${\hat{\rho }}_{\mathrm{mk}|c_{t}}^{*}$, the primary measurement parameter from the previous iteration as

(14) $${\hat{\rho }}_{\mathrm{mk}|c_{t}}=\frac{\sum_{i\in O_{\mathrm{mt}}}\theta_{i(c_{t})}^{\mathrm{obs}}I(Y_{\mathrm{imt}}=k)+\sum_{i\in O_{\mathrm{mt}}^{c}}\theta_{i(c_{t})}^{\mathrm{obs}}{\hat{\rho }}_{\mathrm{mk}|c_{t}}^{*}}{\sum_{i=1}^{N}\theta_{i(c_{t})}^{\mathrm{obs}}}.$$

### 3.2. Inference

The likelihood function of LCPM with *K* latent classes and *S* profiles has K!×S! different parameter values with the same likelihood. This is because the likelihood function is invariant to permutations of class and profile labels. Such a symmetric structure makes the parameters of LCPM unidentifiable. Still, the local identifiability [23] can be established in that one can find an open neighborhood of the parameter such that every parameter in that neighborhood generates a unique distribution [18]. Such local identifiability can be examined by investigating the positive definiteness of the Fisher information at the parameter estimates [13]. In addition, the asymptotic identifiability validates using the Fisher information matrix for inferences [22].

Consequently, we use Fisher's observed information matrix to calculate the asymptotic standard errors of the EM estimates. Specifically, we calculate the second derivative of the logarithm of the observed data likelihood in Equation (6) for all free parameters, evaluate it at MLE, and take its (negative one times) inverse. The square root of the diagonal elements of the information matrix becomes the asymptotic standard error of the EM estimates. The details of the first and the second derivatives of LCPM are in the Appendix. Section 4 demonstrates that the 95% confidence intervals based on this method show reasonable coverage probabilities.

### 3.3. Determining the numbers of latent classes and profiles

When fitting an LCPM, the number of latent classes and profiles [K,S] should be prespecified. The likelihood-ratio test (LRT) statistics cannot be used for comparing different models because two models with different numbers of latent classes are not in nested relationship [8]. Alternatively, we adopted BIC to assess relative model fit among candidate models with different numbers of classes [25]. The model with a smaller BIC is preferred.

As an absolute model fit, we investigate the bootstrap p-value of the selected model to check whether the model is appropriate for data or not. Specifically, we obtain ML estimates of the specified LCPM and evaluate $G_{\mathrm{obs}}^{2}=-2(\log L_{\mathrm{mod}}-\log L_{\mathrm{sat}})$, where $L_{\mathrm{mod}}$ denotes the observed data likelihood of the LCPM and $L_{\mathrm{sat}}$ is the of the saturated model [16,24]. Next, we generate a *b*th bootstrap data set and calculate $G_{\left(b\right)}^{2}$, for b=1…,100. Finally, the bootstrap p-value is calculated as the proportion of $G_{\left(b\right)}^{2}$s that are greater than $G_{\mathrm{obs}}^{2}$. We conclude that the specified LCPM is appropriate for the data if the bootstrap p-value exceeds α=0.05.

## 4. Simulation studies

### 4.1. Inference on LCPM

We present several simulation studies to evaluate the performance of our proposed method, focusing on the point and interval estimation of parameters. We simulate a data set, fit a LCPM and calcuate point and interval estimates. Such a process is repeated for 500 times, and standardized biase (Bias), length of 95% confidence interval (CI), root mean-squared errors (RMSE), and coverage probabilities (CP) are evaluated. Each data set contains a time-dependent covariate $z_{\mathrm{it}}\overset{\mathrm{iid}}{∼}N(1,1)$, for three time periods (*T* = 3), and a time-independent predictor $x_{i}\overset{\mathrm{iid}}{∼}N(1,1)$. We also generate four binary response variables across three time periods (that is, $\left[Y_{1t},\ldots Y_{4t}\right]$, *t* = 1, 2, 3). For each time point, a categorical latent variable $C_{t}$ at time *t* has two classes (*K* = 2). A latent profile variable *U* has two latent profiles (*S* = 2).

We consider four different simulation scenarios in sample size *N* and the class separation. The size of each simulated data is either 250 or 500, where *N* = 250 mimics our real-data set and *N* = 500 represents a large sample scenario. Also, primary measurement parameters are (i) strong ( ρ=0.9 or 0.1), or (ii) mixed (some values are close to 0.5), where the strong measurement parameters generate clear separations between classes, and the mixed parameters yield overlapping classes. Lastly, we assume that primary measurement parameters are time-constant (that is, $\rho_{\mathrm{mk}|c}=P(Y_{\mathrm{mt}}=k|C_{t}=c)$ for all *t* = 1, 2, 3). Table 1 illustrates the true parameters of strong and mixed parameter values

**Table 1.** True parameter values in two different scenarios.

Parameter	Strong	Mixed	Parameter	Strong	Mixed
$\rho_{11|1}$	0.9	0.9	$\alpha_{12|1}^{\left(1\right)}$	log(9)−1	log(9)−1
$\rho_{21|1}$	0.9	0.9	$\alpha_{02|2}^{\left(1\right)}$	−1.0	−1.0
$\rho_{31|1}$	0.9	0.7	$\alpha_{12|2}^{\left(1\right)}$	log(1/9)+1	log(1/9)+1
$\rho_{41|1}$	0.9	0.7	$\alpha_{02|1}^{\left(2\right)}$	1.0	1.0
$\rho_{11|2}$	0.1	0.1	$\alpha_{12|1}^{\left(2\right)}$	log(9)−1	log(9)−1
$\rho_{21|2}$	0.1	0.1	$\alpha_{02|2}^{\left(2\right)}$	−1.0	−1.0
$\rho_{31|2}$	0.1	0.3	$\alpha_{12|2}^{\left(2\right)}$	log(1/9)+1	log(1/9)+1
$\rho_{41|2}$	0.1	0.3	$\alpha_{02|1}^{\left(3\right)}$	1.0	1.0
${\text{β}}_{0|2}$	−1.0−	−1.0−	$\alpha_{12|1}^{\left(3\right)}$	log(9)−1	log(9)−1
${\text{β}}_{1|2}$	1.0	1.0	$\alpha_{02|2}^{\left(3\right)}$	−1.0	−1.0
$\alpha_{02|1}^{\left(1\right)}$	1.0	1.0	$\alpha_{12|2}^{\left(3\right)}$	log(1/9)+1	log(1/9)+1

Tables 2 and 3 illustrate the simulation results under two scenarios. We consider any standardized bias with an absolute value greater than 0.4 and coverage probabilities less than 0.9 unacceptable [10]. In both simulation scenarios, absolute values of the standardized bias are less than 0.4 for all parameters, and all coverage probabilities are close to 0.95. The length of 95% confidence intervals and RMSE decreases as sample size increases for given parameter values. Consequently, we conclude that the proposed EM algorithm yields valid point and interval estimates.

**Table 2.** Simulation results under strong measurement parameters.

	*N* = 500				*N* = 250			
Param	Bias	Length	RMSE	CP	Bias	Length	RMSE	CP
$\rho_{11|1}$	0.034	0.023	0.011	0.952	0.020	0.032	0.016	0.956
$\rho_{21|1}$	−0.107−	0.023	0.011	0.960	0.043	0.032	0.016	0.964
$\rho_{31|1}$	−0.025−	0.023	0.011	0.956	0.026	0.033	0.017	0.948
$\rho_{41|1}$	−0.035−	0.023	0.011	0.944	−0.046−	0.032	0.016	0.928
$\rho_{11|2}$	−0.028−	0.023	0.012	0.936	−0.022−	0.032	0.016	0.916
$\rho_{21|2}$	0.001	0.023	0.011	0.936	−0.066−	0.033	0.017	0.948
$\rho_{31|2}$	0.021	0.023	0.012	0.932	−0.001−	0.032	0.017	0.948
$\rho_{41|2}$	0.033	0.023	0.012	0.932	0.041	0.033	0.017	0.940
$\alpha_{02|1}^{\left(1\right)}$	−0.122−	0.369	0.249	0.936	−0.162−	0.686	0.377	0.964
$\alpha_{12|1}^{\left(1\right)}$	−0.086−	0.528	0.274	0.944	−0.170−	0.790	0.426	0.960
$\alpha_{02|2}^{\left(1\right)}$	0.101	0.464	0.226	0.944	0.165	0.679	0.342	0.964
$\alpha_{12|2}^{\left(1\right)}$	0.054	0.530	0.293	0.956	0.171	0.788	0.429	0.944
$\alpha_{02|1}^{\left(2\right)}$	0.132	0.465	0.235	0.948	−0.100−	0.682	0.339	0.956
$\alpha_{12|1}^{\left(2\right)}$	−0.058−	0.522	0.259	0.964	−0.176−	0.798	0.435	0.968
$\alpha_{02|2}^{\left(2\right)}$	−0.042−	0.466	0.245	0.936	0.052	0.675	0.342	0.936
$\alpha_{12|2}^{\left(2\right)}$	0.017	0.535	0.290	0.936	0.173	0.780	0.438	0.940
$\alpha_{02|1}^{\left(3\right)}$	0.130	0.465	0.233	0.948	−0.130−	0.683	0.389	0.940
$\alpha_{12|1}^{\left(3\right)}$	−0.021−	0.522	0.289	0.952	−0.155−	0.783	0.420	0.948
$\alpha_{02|2}^{\left(3\right)}$	−0.099−	0.461	0.221	0.968	0.133	0.679	0.379	0.944
$\alpha_{12|2}^{\left(3\right)}$	−0.012−	0.520	0.259	0.944	0.138	0.783	0.438	0.952
${\text{β}}_{0|2}$	−0.072−	0.317	0.165	0.928	0.066	0.447	0.223	0.964
${\text{β}}_{1|2}$	0.066	0.243	0.120	0.948	0.015	0.343	0.165	0.948

**Table 3.** Simulation results under mixed measurement parameters.

	*N* = 500				*N* = 250			
Param	Bias	Length	RMSE	CP	Bias	Length	RMSE	CP
$\rho_{11|1}$	−0.067−	0.025	0.014	0.956	−0.069−	0.032	0.016	0.945
$\rho_{21|1}$	−0.004−	0.035	0.018	0.960	−0.001−	0.032	0.016	0.970
$\rho_{31|1}$	0.035	0.035	0.017	0.960	0.083	0.050	0.025	0.930
$\rho_{41|1}$	−0.015−	0.025	0.013	0.956	−0.024−	0.050	0.025	0.930
$\rho_{11|2}$	0.023	0.025	0.013	0.980	0.062	0.032	0.016	0.935
$\rho_{21|2}$	−0.034−	0.035	0.018	0.976	−0.087−	0.033	0.017	0.945
$\rho_{31|2}$	−0.010−	0.035	0.018	0.952	0.001	0.032	0.017	0.950
$\rho_{41|2}$	−0.035−	0.025	0.014	0.964	0.036	0.033	0.016	0.950
$\alpha_{02|1}^{\left(1\right)}$	−0.014−	0.528	0.264	0.948	−0.175−	0.686	0.421	0.970
$\alpha_{12|1}^{\left(1\right)}$	−0.252−	0.622	0.335	0.972	−0.182−	0.790	0.508	0.945
$\alpha_{02|2}^{\left(1\right)}$	0.055	0.517	0.226	0.940	0.158	0.679	0.384	0.970
$\alpha_{12|2}^{\left(1\right)}$	0.108	0.591	0.293	0.976	0.171	0.788	0.492	0.960
$\alpha_{02|1}^{\left(2\right)}$	−0.021−	0.598	0.296	0.968	0.260	0.682	0.409	0.950
$\alpha_{12|1}^{\left(2\right)}$	−0.094−	0.518	0.237	0.980	0.212	0.798	0.506	0.950
$\alpha_{02|2}^{\left(2\right)}$	0.056	0.605	0.331	0.960	−0.219−	0.675	0.379	0.975
$\alpha_{12|2}^{\left(2\right)}$	0.127	0.535	0.272	0.968	−0.116−	0.780	0.544	0.950
$\alpha_{02|1}^{\left(3\right)}$	−0.154−	0.527	0.330	0.948	0.063	0.683	0.398	0.955
$\alpha_{12|1}^{\left(3\right)}$	−0.164−	0.614	0.353	0.952	0.284	0.783	0.478	0.960
$\alpha_{02|2}^{\left(3\right)}$	0.168	0.516	0.262	0.960	0.081	0.679	0.408	0.950
$\alpha_{12|2}^{\left(3\right)}$	0.034	0.592	0.315	0.968	0.126	0.783	0.588	0.965
${\text{β}}_{0|2}$	0.115	0.320	0.156	0.956	−0.122−	0.457	0.232	0.965
${\text{β}}_{1|2}$	−0.010−	0.246	0.121	0.964	0.081	0.349	0.167	0.970

### 4.2. Classification accuracy comparison

In this simulation, we compare the classification accuracy of the LCPM with and without time-dependent covariates. Similar to Section 4.1, we generate 100 datasets with two latent classes (*K* = 2) over three-time periods (*T* = 3) and two latent profiles (*S* = 2) under 16 different scenarios, depending on the sample size (*N* = 250 or 500), the separation of latent classes (strongly separated or overlapped), the inclusion of a time-dependent covariate (YES or NO), and whether the time-dependent model in Equation (4) is correctly specified or not. In the correctly specified scenario, we used the same model for Equation (4) in both data generation and model fit. In the incorrectly specified scenario, we assumed that an analyst misspecified the analysis model in Equation (4); the data is generated under the quadratic function of individual-level covariates, while the training model only contains first-order terms. We divide the data set into training and testing data sets and fit two LCPMs for each training data set, one with time-dependent covariates and the other without covariates. Next, we predict latent classes and profile memberships of testing subjects using two fitted LCPMs and calculate the prediction accuracies. For each subject *i*, we use the maximum probability assignment to predict latent classes and profile memberships.

Table 4 illustrates the classification accuracies of LCPM with and without time-dependent covariates across eight scenarios. When the population is generated from LCPM with time-dependent covariates (denoted as ‘Model = Correct’), the model with time-dependent covariates (denoted as ‘Covariate = YES’) shows noticeably higher classification accuracies of latent profiles and classes for all three time periods than the model without covariates across four different scenarios. Such trends are as expected.

**Table 4.** Classification accuracies of two LCPM under eight different scenarios.

Model	Size	Separation	Covariate	Profile	Class at 1	Class at 2	Class at 3
Correct	500	Strong	YES	0.954	0.986	0.986	0.986
			NO	0.873	0.901	0.901	0.901
		Weak	YES	0.938	0.960	0.960	0.960
			NO	0.873	0.901	0.901	0.902
	250	Strong	YES	0.952	0.986	0.985	0.986
			NO	0.856	0.900	0.894	0.902
		Weak	YES	0.938	0.960	0.960	0.960
			NO	0.873	0.901	0.901	0.902
Incorrect	500	Strong	YES	0.966	0.988	0.987	0.987
			NO	0.910	0.903	0.903	0.899
		Weak	YES	0.966	0.988	0.988	0.988
			NO	0.903	0.903	0.901	0.902
	250	Strong	YES	0.955	0.982	0.984	0.982
			NO	0.889	0.897	0.903	0.903
		Weak	YES	0.918	0.943	0.945	0.947
			NO	0.887	0.895	0.898	0.904

When the population is generated from LCPM with time-dependent covariates with their quadratic terms, similar trends can be found in that LCPM with time-dependent covariates yield higher classification accuracies for latent profiles and classes, compared to the model without covariates. Such trends can be found in the rows denoted as ‘Model = incorrect’.

In addition, we repeated simulation studies under a model without covariates so that the true population distribution is independent of time-dependent covariates (not included in the table). In this case, both models yield comparable classification accuracies because the contributions of covariates on classification are negligible. However, such redundant information did not cause misclassification, so we conclude that including time-dependent covariate improves classification accuracies of latent classes and profile memberships, even if the model in Equation (4) is misspecified.

## 5. Application to head and neck cancer (HNC) data

We employ the proposed LCPM to identify latent trajectory memberships of patients with head and neck cancer (HNC). The HNC data set was collected from 275 patients who received complex treatment modalities, including surgery, chemotherapy, and radiation therapy via six binary indicators [30]. A retrospective chart review was conducted with treatment side effects repeatedly collected from the patient charts. Six binary response variables were used to measure side effects during treatment for HNC. Patients were tracked for six weeks via REDCap, and their responses to survey items were recorded.

Table 5 illustrates six binary questionnaire variables related to unpleasant symptoms related to HNC treatment. Also, Table 6 illustrates the sample proportions of patients who responded ‘Yes’ to each binary variable. Finally, we consider several demographic factors as well as time-dependent covariates and time-independent covariates. Descriptions of time-independent and dependent predictors are on Tables A1 and A2 in the Appendix, respectively.

**Table 5.** Descriptions on six binary outcome variables for measuring unpleasant symptoms.

Indicator	Description
Pain	Did the patient report pain?
Mucositis	Did the patient report mucositis?
Taste.alt.presence	Did the patient report taste alterations?
Dry.mouth.presence	Did the patient report dry mouth?
Dysphagia	Did the patient report dysphagia?
Fatigue	Did the patient report fatigue?

**Table 6.** Proportion of patients who responded ‘Yes’ to each binary outcome variable over six time periods.

	Proportion of ‘Yes’ ( %)					
Indicator	Week 1	Week 2	Week 3	Week 4	Week 5	Week 6
Pain	37.8%	48.4%	65.5%	70.9%	73.5%	77.1%
Mucositis	09.1%	40.4%	72.0%	85.8%	92.4%	96.7%
Taste.alt.presence	24.4%	50.5%	70.2%	82.9%	89.8%	93.1%
Dry.mouth.presence	25.5%	52.4%	76.0%	88.4%	0.942	99.3%
Dysphagia	68.4%	82.5%	91.6%	98.2%	98.9%	99.6%
Fatigue	37.5%	60.4%	73.8%	85.8%	91.3%	95.3%

### 5.1. Model selection

As discussed in Section 3.3, the model selection procedure in LCPM might entail tedious trials and errors because the number of latent class *K* and the number of latent profile *S* freely vary. We range the number of latent classes from 2 to 6 and the number of latent profiles from 2 to 6. In such a way, we investigate 25 models that are different in the number of latent classes and latent profiles.

It is known that the model selection procedure in the latent class model can be done without considering the covariate effects due to the marginalization property [3,16,19]. In this sense, the covariate effects are excluded during the model selection step. In addition, each model is repeatedly fitted for 100 times with varying initial values to ensure that the model parameters are locally identifiable and the solution does not converge to local maxima.

Table 7 shows the estimated BIC values of LCPM with different numbers of latent classes/profiles. The model with two classes and three profiles has the smallest BIC value among all candidates. However, the bootstrap p-values of the two-class models are all less than 0.01, which implies that two-class models are not appropriate for the data. We consider the model with three latent classes and three latent profiles. The bootstrap p-value of the model is 0.58, and the corresponding BIC value is the smallest among all candidates with three or more latent classes.

**Table 7.** BIC values of LCPM with different numbers of latent classes/profiles.

	Profile				
Class	2	3	4	5	6
2	1778.48	1744.70	1768.43	1794.26	1798.82
3	1899.25	1879.05	1936.23	1995.80	2063.97
4	1980.03	2013.42	2102.69	2197.35	2300.05
5	2080.80	2147.78	2270.04	2398.91	2536.36
6	2181.57	2282.14	2438.56	2600.44	2769.44

Once the number of latent classes and profiles are selected, we investigate whether the primary measurement parameters are equal across all time periods; this is equivalent to test $H_{0}:\rho_{\mathrm{mk}|c_{1}}=\cdots \rho_{\mathrm{mk}|c_{6}},\mathrm{\forall m},k$ is not rejected or not. We fit two LCPM with three classes and profiles (one under $H_{0}$ and another without constraints) and implement the likelihood ratio (LR) statistics. The two maximized likelihoods are almost identical, and the corresponding p-value is close to 1. Consequently, we adopt the *ρ*-constrained three-class, three-profile model as the final model. Finally, we rerun the three-class, three-profile models with time independent and dependent covariates.

### 5.2. Parameter estimates

Table 8 illustrates estimated primary measurement parameters (i.e. *ρ* parameters) of three latent classes. As introduced in Section 2, each primary measurement parameter denotes the probability of ‘Yes’ to each binary item given latent class memberships. The first latent class represents a group of patients who are less likely to suffer from side effects except for ‘Dysphagia’. In this sense, the first class can be labeled as the ‘mild-symptom’ class (*Mild*). Similarly, the second latent class can be labeled as the ‘moderate-symptom’ group (*Moderate*) because patients in this latent class are more likely to experience symptoms compared to people in the *Mild* class. In a similar manner, the third class represents the ‘severe-symptom’ class (*Severe*) because patients in this class are highly likely to experience symptoms. Finally, the proportions of the three classes over time are illustrated in the last six rows of Table 8. As time passes, the proportion of *Mild* class decreases monotonously, while the proportion of *Severe* gradually increases.

**Table 8.** Primary measurement parameter estimates for three classes and their proportions.

Binary indicator	Class 1 (*Mild*)	Class 2 (*Moderate*)	Class 3 (*Severe*)
Pain	0.241	0.619	0.811
Mucositis	0.080	0.589	1.000
Taste.alt.presence	0.232	0.568	0.997
Dry.mouth.presence	0.167	0.727	1.000
Dysphagia	0.617	0.950	1.000
Fatigue	0.324	0.736	0.948
Time	Latent class prevalence		
Week 1	0.792	0.208	0.000
Week 2	0.435	0.456	0.110
Week 3	0.066	0.638	0.297
Week 4	0.000	0.379	0.621
Week 5	0.000	0.235	0.765
Week 6	0.000	0.121	0.879

Table 9 illustrates the secondary measurement parameters (i.e. *η* parameters) of three latent profiles. The first latent profile, which takes 23.5% of the sample, represents a group of patients who are likely to belong to *Mild* class at Week 1, then transit to *Moderate* class after Week 3. The second latent profile, which takes 45.3% of the sample, represents a group of patients who are likely to belong to *Mild* class at Week 1, then gradually transit to *Moderate* class by Week 3, and end up to *Severe* class after Week 4. In this sense, we can distinguish patients in *Profile 2* from *Profile 1* via latent trajectories from Week 4 to 6. We find that *Profile 2* represents dramatic transitions from *Mild* to *Severe* class during the second, third, and fourth week of treatment, which implies that patients who belong to *Profile 2* are likely to suffer from symptom worsening. Finally, *Profile 3* represents a group of patients whose major latent class membership is *Moderate*, ending up in *Severe* by Week 3. This profile takes 31.2% of the sample. Such results clearly illustrate the advantage of employing entire sets of survey responses using LCPM. By doing so, we can scrutinize the sequential patterns of three latent classes for all time points and identify the representative groups of the sample (i.e. latent profile) based on the patterns of the three latent classes.

**Table 9.** Secondary measurement parameter estimates for three profiles and their proportions.

Profile	Class	Week 1	Week 2	Week 3	Week 4	Week 5	Week 6
	*Mild*	0.965	0.713	0.279	0.000	0.000	0.000
*Profile 1* ( 23.5%)	*Moderate*	0.035	0.287	0.721	1.000	1.000	0.514
	*Severe*	0.000	0.000	0.000	0.000	0.000	0.486
	*Mild*	1.000	0.589	0.000	0.000	0.000	0.000
*Profile 2* ( 45.3%)	*Moderate*	0.000	0.411	1.000	0.317	0.000	0.000
	*Severe*	0.000	0.000	0.000	0.683	1.000	1.000
	*Mild*	0.360	0.000	0.000	0.000	0.000	0.000
*Profile 3* ( 31.2%)	*Moderate*	0.640	0.649	0.049	0.000	0.000	0.000
	*Severe*	0.000	0.351	0.951	1.000	1.000	1.000

Table 10 illustrates the estimated associations between time-independent predictors and latent profile memberships. As introduced in Equation (3), β denotes a vector of regression coefficient that depicts associations between patient-level time-independent predictors and latent profile memberships. Standard errors of the estimated regression coefficients are obtained by inverting the Hessian matrix of the model evaluated at the parameter estimates. We transform regression coefficients from multinomial logistic regression into odds ratios for better interpretations, where *Profile 1* is the baseline profile. In such a way, each estimated odds ratio represents the association between latent profile membership and a predictor variable. For example, the odds of belonging to *Profile 3* compared to *Profile 1* increases by 1.867 times (with 95% CI: [1.236,2.822]) as *Cancer stage* increases by 1 unit.

**Table 10.** Estimated odds ratios of time-independent covariates and their 95% confidence intervals.

Variable	*Profile 2*	*Profile 3*
HPV	0.555 [0.215, 1.430]	0.705 [0.287, 1.729]
Age	0.996 [0.965, 1.028]	0.982 [0.952, 1.013]
Gender	2.046 [0.872, 4.798]	0.924 [0.404, 2.109]
Cancer Stage	1.132 [0.780, 1.644]	1.867 [1.236, 2.822]
Alcohol Use	1.436 [0.616, 3.348]	1.261 [0.535, 2.975]
Smoking	1.104 [0.483, 2.520]	0.945 [0.410, 2.176]
Lives Alone	0.469 [0.199, 1.107]	0.661 [0.270, 1.620]

Finally, we investigate associations between time-dependent covariates and latent class memberships conditioning on latent profile memberships. As introduced in Equation (4), $\alpha_{c_{t}|u}^{\left(t\right)}$ denotes a vector of regression coefficient that depicts associations between patient-specific time-dependent covariates at Week *t* and latent class memberships $c_{t}$, conditioning on their latent profile memberships *u*, where *u* = 1, 2, 3. The regression coefficients for Week 4, 5 for *Profile 1*, Week 1, 3, 5, 6 for *Profile 2*, and Week 4, 5, and 6 for *Profile 3* are not identifiable due to the boundary solutions (that is, some latent classes have very small proportions). In this case, we constrained coefficients of covariates to be 0 and only estimated intercept. For brevity, we only report estimated associations that are statistically significant at a significant level α=0.05. Odds of belonging to *Moderate* class compared to *Mild* increases by 1.061 (with 95% CI: [1.005,1.121]) times as *HGB* increases by one unit at Week 2, if a patient belongs to *Profile 2*. Also, odds of belonging to *Moderate* class (vs *Mild*) decreases by 0.967 (with 95% CI: [0.936,0.998]) times as *WT* increases by one unit at Week 1, if a patient belongs to *Profile 3*.

## 6. Discussion

This paper proposes an extended version of LCPM by embedding time-dependent covariates on the class prevalence parameters. The proposed LCPM contains time-varying predictors and thus allows analysts to investigate the association between predictors and estimated latent class memberships via multinomial logistic regressions. Given response patterns and covariate values, the estimated regression coefficients provide the conditional probabilities of latent classes and profile memberships. In such a way, our model offers patient-level interpretations and enhances classification accuracies on latent classes and profile memberships.

In this paper, we suggest using a one-step estimation, which simultaneously estimates regression coefficients and measurement parameters using the EM algorithm. The proposed method avoids potential biases due to misclassification of latent class/profile membership, which often occurs in three-step approaches. To promote our methodology, we have written a program to implement LCPM in the R language (version 3.6.1) available at https://sites.google.com/site/leejwegg/ or upon request.

As a real data example, we analyze the HNC data set using our proposed model and discover novel insights about HNC patients who underwent CT and RT concurrently. Owing to the smaller number of free parameters and the two-level latent structure, our proposed model successfully incorporates survey responses from all time points and discovers three latent classes and profiles. In such a way, the proposed LCPM may provide better insights into the symptom patterns of patients who experienced both CT and RT over six weeks. We hope our model is widely adopted for longitudinal analyses with multivariate outcomes, especially for categorical outcomes with missing values.

For a given convergence criterion of the EM algorithm, the computational time for a single fit of the proposed LCPM depends on the sample size, the numbers of categorical outcome variables and predictors, and the complexity of the model structure (i.e. the numbers of latent classes and profiles). While a single implementation of LCPM with three latent classes and three profiles on six binary outcome variables takes less than a minute, repeating an LCPM fit with different initial values may take up to several hours. Note that this is not a disadvantage of LCPM but a fundamental challenge of the EM algorithm, whose convergence to the global maximum is not guaranteed if an inappropriate initial value is given.

Lastly, we discuss two future research directions. The framework of variable selection in LCPM should be established to deal with a large number of covariates that are of interest. We believe adding a penalization term on the objective function (in our case, the expected complete data likelihood) may help to shrink the dimension of nonzero regression coefficients. We are investigating the shrinkage estimation under the latent variable framework. Another interesting topic is missing values in patient-level covariates. For example, researchers have proposed Bayesian inferences for missing covariates by embedding prior information on covariate distributions and missing mechanisms under various frameworks, such as survival analysis or generalized linear models. For more details, see Huang *et al*. [14], Ibrahim *et al*. [15] and their references. Similarly, developing a strategy for missing covariates under latent variable modeling is highly needed.

## Appendix

### A.1. The first derivative of the LCPM

Equation (A1) shows the elements of first derivatives with respect to parameters [ρ,α,β].

(A1)
$$\begin{array}{rl} \frac{\partial \mathrm{logL}}{\partial \rho_{\mathrm{mk}|c_{t}}^{\left(t\right)}} & =\sum_{i=1}^{N}\frac{\theta_{i(c_{t})}I(Y_{\mathrm{imt}}=k)}{\rho_{\mathrm{mk}|c_{t}}^{\left(t\right)}},\\ \frac{\partial \mathrm{logL}}{\partial {\text{β}}_{\mathrm{pu}}} & =\sum_{i=1}^{N}x_{\mathrm{ip}}(\theta_{i(u)}-\gamma_{u}(x_{i})),\\ \frac{\partial \mathrm{logL}}{\partial \alpha_{qc_{t}|u}^{\left(t\right)}} & =\sum_{i=1}^{N}z_{\mathrm{iqt}}(\theta_{i(u,c_{t})}-\eta_{c_{t}|u}^{\left(t\right)}(z_{\mathrm{it}})\theta_{i(u)}). \end{array}$$

Here, we have $k=1,\ldots ,r_{m}$, m=1,…,M, $c_{t}=1\ldots K$, t=1,…,T, u=1,…,S, p=1,…,P, and q=1,…,Q. Also, the measurement parameters ρ are constrained in a way that $\sum_{k=1}^{r_{m}}\rho_{\mathrm{mk}|c_{t}}^{\left(t\right)}=1$ for all subscripts.

### A.2. The second derivative of the LCPM

This section provides details of the second derivative matrix with respect to [ρ,α,β]. The second derivatives with respect to parameters ρ and the others are given as

(A2)
$$\begin{array}{rl} \frac{\partial^{2}\mathrm{logL}}{\partial \rho_{\mathrm{mk}|c_{t}}^{\left(t\right)}\partial \rho_{m^{\prime }k^{\prime }|c_{t^{\prime }}^{\prime }}^{\left(t^{\prime }\right)}} & =\sum_{i=1}^{N}\frac{\theta_{i(c_{t},c_{t^{\prime }}^{\prime })}(1-\zeta_{tt^{\prime }})+\theta_{i(c_{t})}\left\{\zeta_{tt^{\prime }}(1-\zeta_{c_{t}c_{t^{\prime }}^{\prime }})+(1-\zeta_{mm^{\prime }})-\theta_{i(c_{t^{\prime }}^{\prime })}\right\}}{\rho_{\mathrm{mk}|c_{t}}\rho_{m^{\prime }k^{\prime }|c_{t^{\prime }}}},\\ \frac{\partial^{2}\mathrm{logL}}{\partial \rho_{\mathrm{mk}|c_{t}}^{\left(t\right)}\partial \alpha_{qc_{t^{\prime }}^{\prime }|u}^{\left(t^{\prime }\right)}} & =\sum_{i=1}^{N}\frac{z_{\mathrm{iq}t^{\prime }}\left\{\theta_{i(u,c_{t},c_{t^{\prime }}^{\prime })}(1-\zeta_{tt^{\prime }})+\theta_{i(u,c_{t^{\prime }}^{\prime })}(\zeta_{c_{t}c_{t^{\prime }}^{\prime }}-\theta_{i(c_{t})})-\eta_{c_{t^{\prime }}^{\prime }}\theta_{i(u,c_{t})}\right\}\zeta_{Y_{\mathrm{imt}}k}}{\rho_{\mathrm{mk}|c_{t}}^{\left(t\right)}},\\ \frac{\partial^{2}\mathrm{logL}}{\partial \rho_{\mathrm{mk}|c_{t}}^{\left(t\right)}\partial {\text{β}}_{\mathrm{pu}}} & =\sum_{i=1}^{N}\frac{x_{\mathrm{ip}}\left\{\theta_{i(u,c_{t})}-\theta_{i(u)}\theta_{i(c_{t})})\zeta_{Y_{\mathrm{imt}}k}\right\}}{\rho_{\mathrm{mk}|c_{t}}^{\left(t\right)}},\\ \frac{\partial^{2}\log L}{\partial {\text{β}}_{\mathrm{pu}}\partial {\text{β}}_{p^{\prime }u^{\prime }}} & =\sum_{i=1}^{N}x_{\mathrm{ip}}x_{ip^{\prime }}\left\{\theta_{i(u)}(\zeta_{uu^{\prime }}-\theta_{i(u^{\prime })})-\gamma_{u}(x_{i})(\zeta_{uu^{\prime }}-\gamma_{u^{\prime }}(x_{i}))\right\},\\ \frac{\partial^{2}\log L}{\partial {\text{β}}_{\mathrm{pu}}\partial \alpha_{qc_{t}|u^{\prime }}^{\left(t\right)}} & =\sum_{i=1}^{N}x_{\mathrm{ip}}\left\{\frac{\partial \theta_{i(u)}}{\partial \alpha_{qc_{t}|u^{\prime }}^{\left(t\right)}}-\frac{\partial \gamma_{u}(x_{i})}{\partial \alpha_{qc_{t}|u^{\prime }}^{\left(t\right)}}\right\},\\ \frac{\partial^{2}\log L}{\partial \alpha_{qc_{t}|u}^{\left(t\right)}\partial \alpha_{q^{\prime }c_{t^{\prime }}^{\prime }|u^{\prime }}^{\left(t^{\prime }\right)}} & =\sum_{i=1}^{N}z_{\mathrm{iqt}}\left\{\frac{\partial \theta_{i(u,c_{t})}}{\partial \alpha_{q^{\prime }c_{t^{\prime }}^{\prime }|u^{\prime }}^{\left(t^{\prime }\right)}}-\frac{\partial \eta_{c_{t}|u}^{\left(t\right)}(z_{\mathrm{it}})}{\partial \alpha_{q^{\prime }c_{t^{\prime }}^{\prime }|u^{\prime }}^{\left(t^{\prime }\right)}}\theta_{i(u)}-\eta_{c_{t}|u}^{\left(t\right)}(z_{\mathrm{it}})\frac{\partial \theta_{i(u)}}{\partial \alpha_{q^{\prime }c_{t^{\prime }}^{\prime }|u^{\prime }}^{\left(t^{\prime }\right)}}\right\}, \end{array}$$

with derivatives of posterior probabilities with respect to α are given as

(A3)
$$\begin{array}{r} \begin{array}{rl} \frac{\partial \eta_{c_{t}|u}^{\left(t\right)}(z_{\mathrm{it}})}{\partial \alpha_{q^{\prime }c_{t^{\prime }}^{\prime }|u^{\prime }}^{\left(t^{\prime }\right)}} & =z_{iq^{\prime }t^{\prime }}\eta_{c_{t}|u}^{\left(t\right)}(z_{\mathrm{it}})\left\{\zeta_{c_{t}c_{t^{\prime }}^{\prime }}-\eta_{c_{t^{\prime }}^{\prime }|u^{\prime }}^{\left(t\right)}(z_{it^{\prime }})\right\}\zeta_{uu^{\prime }}\zeta_{tt^{\prime }},\\ \frac{\partial \theta_{i(u)}}{\partial \alpha_{q^{\prime }c_{t^{\prime }}^{\prime }|u^{\prime }}^{\left(t^{\prime }\right)}} & =z_{iq^{\prime }t^{\prime }}(\zeta_{uu^{\prime }}-\theta_{i(u)})\left\{\theta_{i(u^{\prime },c_{t^{\prime }}^{\prime })}-\eta_{c_{t^{\prime }}^{\prime }|u^{\prime }}^{\left(t^{\prime }\right)}(z_{it^{\prime }})\theta_{i(u^{\prime })}\right\},\\ \frac{\partial \theta_{i(u,c_{t})}}{\partial \alpha_{q^{\prime }c_{t^{\prime }}^{\prime }|u^{\prime }}} & =z_{iq^{\prime }t^{\prime }}\theta_{i(u,c_{t})}\\ & \;\times \left\{\zeta_{uu^{\prime }}\left\{(1-\zeta_{tt^{\prime }})\theta_{i(u,c_{t},c_{t^{\prime }}^{\prime })}+\zeta_{c_{t}c_{t^{\prime }}^{\prime }}-\eta_{c_{t^{\prime }}^{\prime }|u^{\prime }}(z_{it^{\prime }})\right\}-\theta_{i(u^{\prime },c_{t^{\prime }}^{\prime })}+\eta_{c_{t^{\prime }}^{\prime }|u^{\prime }}^{\left(t^{\prime }\right)}(z_{it^{\prime }})\theta_{i(u^{\prime })}\right\}. \end{array} \end{array}$$

In Equations (A2) and (A3), subscript ranges are $q=1,\ldots ,K,h=0,1,k=1,\ldots ,r_{p},p=1,\ldots ,P,m=1,\ldots ,P,u=1\ldots S,w=1\ldots D$, and $\zeta_{ww^{\prime }}$ is defined as 1 if $w=w^{\prime }$ and 0 if $w\neq w^{\prime }$. Note that $\gamma_{u}(x_{i})$ is a constant to α because it is a function of β only. Consequently, the $\gamma_{u}(x_{i})$ derivative with respect to $\alpha_{c_{t}|u}^{\left(t\right)}$ is 0 for all subscripts.

### A.3. Summary of patient-level covariates

**Table A1.** Time-independent patient-level covariates.

Variable	Description
Age	Age in years
Gender	1 if male and 0 if female
HPV	1 if human papillomavirus status (HPV) of tumor is positive and 0 if not
Cancer Stage	Stage of cancer, 0, 1, 2, or 3
Alcohol Use	1 if current drinker, 0 if not a current drinker, and 2 if former drinker
Smoking	1 if current smoker, 0 if not a smoker, and 2 if former smoker
Lives Alone	1 if patient lives alone and 0 if not

**Table A2.** Time-dependent patient-level biomarkers.

Biomarkers	Description
WT	Weight (kilograms) measured at each visit
WBC	White blood cell count (K/uL) measured at each visit
RBC	Red blood cell count (Mil/dL) measured at each visit
HGB	Hemoglobin (g/dL) measured at each visit
Hematocrit	Hematocrit ( %) measured at each visit
MVC	Mean corpuscular volume (fL) measured at each visit
ANC	Absolute neutrophil count (K/uL) measured at each visit
